# A mathematical model for the novel coronavirus epidemic in Wuhan, China

**DOI:** 10.3934/mbe.2020148

**Published:** 2020-03-11

**Authors:** Chayu Yang, Jin Wang

**Affiliations:** Department of Mathematics, University of Tennessee at Chattanooga, 615 McCallie Ave., Chattanooga, TN 37403, USA

**Keywords:** COVID-19, compartmental modeling, basic reproduction number

## Abstract

We propose a mathematical model to investigate the current outbreak of the coronavirus disease 2019 (COVID-19) in Wuhan, China. Our model describes the multiple transmission pathways in the infection dynamics, and emphasizes the role of the environmental reservoir in the transmission and spread of this disease. Our model also employs non-constant transmission rates which change with the epidemiological status and environmental conditions and which reflect the impact of the on-going disease control measures. We conduct a detailed analysis of this model, and demonstrate its application using publicly reported data. Among other findings, our analytical and numerical results indicate that the coronavirus infection would remain endemic, which necessitates long-term disease prevention and intervention programs.

## 1. Introduction

A severe outbreak of respiratory illness started in Wuhan, a city of 11 million people in central China, in December 2019. The causative agent is the novel coronavirus which was identified and isolated from a single patient in early January and subsequently verified in 16 additional patients [1]. The virus is believed to have a zoonotic origin. In particular, the Huanan Seafood Market, a live animal and seafood wholesale market in Wuhan, was regarded as a primary source of this epidemic, as it is found that 55% of the first 425 confirmed cases were linked to the marketplace [2]. Meanwhile, recent comparisons of the genetic sequences of this virus and bat coronaviruses show a 96% similarity [3]. This is the third zoonotic human coronavirus emerging in the current century, after the severe acute respiratory syndrome coronavirus (SARS-CoV) in 2002 that spread to 37 countries and the Middle East respiratory syndrome coronavirus (MERS-CoV) in 2012 that spread to 27 countries. Typical symptoms of COVID-19 infection include dry cough, fever, fatigue, breathing difficulty, and bilateral lung infiltration in severe cases, similar to those caused by SARS-CoV and MERS-CoV infections [4]. Some people also develop non-respiratory symptoms such as nausea, vomiting, and diarrhea [5, 6].

The COVID-19 outbreak is currently on-going and the number of infections has been fast growing since the onset of the epidemic. By January 23, 571 confirmed cases had been reported in China and the infections were already exported to the US, Thailand, Japan and Republic of Korea [7, 8]. As a bold effort to contain the epidemic, the Chinese government ordered to lock down Wuhan on January 23 and at least 15 other cities in the following days, effectively restricting the movement of more than 50 million people in central China, which is considered as the largest quarantine in human history. As of February 10, more than 42,600 people in China have been diagnosed with coronavirus [7]. On January 30, the World Health Organization (WHO) formally declared the outbreak of novel coronavirus a Global Public Health Emergency of International Concern [9].

Several factors complicate the infection dynamics of COVID-19 and add challenges to the disease control. First, the origin of the infection is still uncertain, although it is widely speculated that wild animals such as bats, civets and minks are responsible for starting the epidemic [3]. Second, clinical evidence shows that the incubation period of this disease ranges from 2 to 14 days. During this period of time, infected individuals may not develop any symptoms and may not be aware of their infection, yet they are capable of transmitting the disease to other people [10]. Third, the virus is new and there are no antiviral drugs or vaccines currently available. Consequently, disease control heavily relies on prompt detection and isolation of symptomatic cases. Additionally, the disease outbreak took place right before the Spring Festival (i.e., the Lunar New Year), the most important holiday in China, and a huge population (several millions for the city of Wuhan alone, and hundreds of millions on a national scale) traveled during the month of January 2020, which makes fast and wide spread of the infection possible.

A number of modeling studies have already been performed for the COVID-19 epidemic. Wu et al. [11] introduced a susceptible-exposed-infectious-recovered (SEIR) model to describe the transmission dynamics, and forecasted the national and global spread of the disease, based on reported data from December 31, 2019 to January 28, 2020. They also estimated that the basic reproductive number for COVID-19 was about 2.68. Read et al. [12] reported a value of 3.1 for the basic reproductive number based on data fitting of a SEIR model, using an assumption of Poisson-distributed daily time increments. Tang et al. [13] proposed a deterministic compartmental model incorporating the clinical progression of the disease, the individual epidemiological status, and the intervention measures. They found that the control reproductive number could be as high as 6.47, and that intervention strategies such as intensive contact tracing followed by quarantine and isolation can effectively reduce the control reproduction number and the transmission risk. Imai et al. [14] conducted computational modeling of potential epidemic trajectories to estimate the size of the disease outbreak in Wuhan, with a focus on the human-to-human transmission. Their results imply that control measures need to block well over 60% of transmission to be effective in containing the outbreak. In addition, Gao et al. [15] developed a deep learning algorithm to analyze the infectivity of the novel coronavirus and predict its potential hosts. Their results indicate that bats and minks may be two animal hosts of this virus.

Most of these models have emphasized the significant role of the direct, human-to-human transmission pathway in this epidemic [16], as highlighted by the facts that the majority of the infected individuals did not have any contact with the marketplaces in Wuhan, that the number of infections has been rapidly increasing, and that the disease has spread to all provinces in China as well as more than 20 other countries. In particular, a large number of infected individuals exhibit a relatively long incubation period so that they do not show any symptoms and are unaware of their infection for as long as 10–14 days, during which time they can easily transmit the disease to other people through direct contact.

On the other hand, the models published thus far have not taken into account the role of the environment in the transmission of COVID-19. For example, it is reported that environmental samples taken from the areas of the Huanan Seafood Market have come back positive for the novel coronavirus [4], suggesting that the pathogen may be transmitted through the environmental reservoir. When infected individuals cough or sneeze, they may spread the virus to the environment through their respiratory droplets which then may go on to infect other people with close contact of the same area. Such transmission would especially be facilitated during the early period of the disease outbreak when the general public was not aware of the infection risk, infected individuals were not isolated, and most people did not wear face masks. Even worse, there is a possibility that the virus may survive in the environment for several days, increasing the risk of contamination via surfaces and fomites [17, 18]. Such environmental survival was confirmed for SARS-CoV [19]. A most recent study, based on the review of 22 types of coronaviruses, reveals that coronaviruses such as SARS-CoV, MERS-CoV and endemic human coronaviruses can persist on inanimate surfaces like metal, glass or plastic for up to 9 days [20], providing strong evidences for the pathogen’s environmental survival. Additionally, the novel coronavirus has been found in the stool of some infected individuals [5], which may contaminate the aquatic environment, and fecal-oral contact remains a possible route of transmission for this disease.

In the present paper, we present a new mathematical model for COVID-19 that incorporates multiple transmission pathways, including both the environment-to-human and human-to-human routes. In particular, we introduce an environmental compartment that represents the pathogen concentration in the environmental reservoir. A susceptible individual may contract the disease through the interaction with the contaminated environment, with an infectious but asymptomatic individual, or with an infectious and symptomatic individual. Meanwhile, the transmission rates in our model depend on the epidemiological status and environmental conditions which change with time. In particular, when the infection level is high, people would be motivated to take necessary action to reduce the contact with the infected individuals and contaminated environment so as to protect themselves and their families, leading to a reduction of the average transmission rates. Such varied transmission rates also reflect the strong disease control measures that the Chinese government has implemented, including large-scale quarantine, intensive tracking of movement and contact, strict isolation, extending the Lunar New Year holiday, and advising the public to stay home and avoid spreading infection.

The remainder of this paper is organized as follows. In Section 2, we present our model and assumptions, and conduct a detailed mathematical analysis. In Section 3, we conduct numerical simulation by incorporating the infection data reported for the city of Wuhan. In Section 4, we conclude the paper with some discussion.

## 2. Model formulation and analysis

### 2.1. Formulation

We divide the total human population into four compartments: the susceptible (denoted by *S*), the exposed (denoted by *E*), the infected (denoted by *I*), and the recovered (denoted by *R*). Individuals in the infected class have fully developed disease symptoms and can infect other people. Individuals in the exposed class are in the incubation period; they do not show symptoms but are still capable of infecting others. Thus, another interpretation of the *E* and *I* compartments in our model is that they contain asymptomatic infected and symptomatic infected individuals, respectively.

We introduce the following model to describe the transmission dynamics of the COVID-19 epidemic:

(2.1) $$\frac{dS}{dt}=\Lambda -\beta_{E}(E)SE-\beta_{I}(I)SI-\beta_{V}(V)SV-\mu S,\;\frac{dE}{dt}=\beta_{E}(E)SE+\beta_{I}(I)SI+\beta_{V}(V)SV-(\alpha +\mu )E,\;\frac{dI}{dt}=\alpha E-(w+\gamma +\mu )I,\;\frac{dR}{dt}=\gamma I-\mu R,\;\frac{dV}{dt}=\xi_{1}E+\xi_{2}I-\sigma V,$$

where *V* is the concentration of the coronavirus in the environmental reservoir. The parameter Λ represents the population influx, *μ* is the natural death rate of human hosts, *α*^−1^ is the incubation period between the infection and the onset of symptoms, *w* is the disease-induced death rate, *γ* is the rate of recovery from infection, *ξ*_1_ and *ξ*_2_ are the respective rates of the exposed and infected individuals contributing the coronavirus to the environmental reservoir, and *σ* is the removal rate of the virus from the environment. The functions *β*_*E*_(*E*) and *β*_*I*_(*I*) represent the direct, human-to-human transmission rates between the exposed and susceptible individuals, and between the infected and susceptible individuals, respectively, and the function *β*_*V*_(*V*) represents the indirect, environment-to-human transmission rate. We assume that *β*_*E*_(*E*), *β*_*I*_(*I*) and *β*_*V*_(*V*) are all non-increasing functions, given that higher values of *E*, *I* and *V* would motivate stronger control measures that could reduce the transmission rates. Specifically, we make the following assumptions:

(A1) *β*_*E*_(*E*), *β*_*I*_(*I*), *β*_*V*_(*V*) are all positive; and
(A2) ${\beta^{\prime }}_{E}\left(E\right)\leq 0$, ${\beta^{\prime }}_{I}\left(I\right)\leq 0$, ${\beta^{\prime }}_{V}\left(V\right)\leq 0$.

Apparently, system (2.1) has a unique disease-free equilibrium (DFE) at

(2.2) $$X_{0}=\left(S_{0},E_{0},I_{0},R_{0},V_{0}\right)=\left(\frac{\Lambda }{\mu },0,0,0,0\right).$$

The infection components in this model are *E*, *I*, and *V*. The new infection matrix *F* and the transition matrix *V* are given by

(2.3) $$F=\left[\begin{array}{ccc} \beta_{E}(0)S_{0} & \beta_{I}(0)S_{0} & \beta_{V}(0)S_{0}\\ 0 & 0 & 0\\ 0 & 0 & 0 \end{array}\right]\text{ and }V=\left[\begin{array}{ccc} \alpha +\mu & 0 & 0\\ -\alpha & w_{1} & 0\\ -\xi_{1} & -\xi_{2} & \sigma \end{array}\right],$$

where *w*_1_ = *w* + *γ* + *μ*. The basic reproduction number of model (2.1) is then defined as the spectral radius of the next generation matrix *FV*^−1^ [21]; i.e.,

(2.4) $$R_{0}=\rho \left(FV^{-1}\right)=\frac{\beta_{E}(0)S_{0}}{\alpha +\mu }+\frac{\alpha \beta_{I}(0)S_{0}}{w_{1}(\alpha +\mu )}+\frac{\left(w_{1}\xi_{1}+\alpha \xi_{2}\right)\beta_{V}(0)S_{0}}{\sigma w_{1}(\alpha +\mu )}\;≔R_{1}+R_{2}+R_{3},$$

which provides a quantification of the disease risk. The first two parts $R_{1}$ and $R_{2}$ measure the contributions from the human-to-human transmission routes (exposed-to-susceptible and infected-to-susceptible, respectively), and the third part $R_{3}$ represents the contribution from the environment-to-human transmission route. These three transmission modes collectively shape the overall infection risk for the COVID-19 outbreak.

### 2.2. Equilibrium analysis

We now analyze the equilibria of the system (2.1) which will provide essential information regarding the long-term dynamics of the disease. Let (*S*, *E*, *I*, *R*, *V*) be an equilibrium of model (2.1) and thereby satisfy the following equations

(2.5) $$\Lambda -\beta_{E}(E)SE-\beta_{I}(I)SI-\beta_{V}(V)SV-\mu S=0,\;\beta_{E}(E)SE+\beta_{I}(I)SI+\beta_{V}(V)SV-(\alpha +\mu )E=0,\;\alpha E-w_{1}I=0,\;\gamma I-\mu R=0,\;\xi_{1}E+\xi_{2}I-\sigma V=0.$$

Solving (2.5) yields

(2.6) $$S=\frac{1}{\mu }(\Lambda -(\alpha +\mu )E),\;E=\frac{w_{1}}{\alpha }I,\;R=\frac{\gamma }{\mu }I,\;V=\frac{w_{1}\xi_{1}+\alpha \xi_{2}}{\sigma \alpha }I.$$

It follows from the first two equations of (2.6) that *S* can be denoted by a function of *I*, namely,

(2.7) $$S=\phi (I)≔\frac{1}{\mu }\left(\Lambda -\frac{w_{1}(\alpha +\mu )}{\alpha }I\right).$$

Meanwhile, in view of the second equation of (2.5) and Eqs (2.6), we obtain

(2.8) $$S=\psi (I)≔(\alpha +\mu ){\left(\beta_{E}\left(\frac{w_{1}}{\alpha }I\right)+\frac{\alpha }{w_{1}}\beta_{I}(I)+\frac{w_{1}\xi_{1}+\alpha \xi_{2}}{\sigma w_{1}}\beta_{V}\left(\frac{w_{1}\xi_{1}+\alpha \xi_{2}}{\sigma \alpha }I\right)\right)}^{-1}.$$

Let us now consider curves *S* = *ϕ*(*I*), *I* ≥ 0 and *S* = *ψ*(*I*), *I* ≥ 0. In particular, the intersections of these two curves in ${\mathbb{R}}_{+}^{2}$ determine the non-DFE equilibria. Clearly, *ϕ*(*I*) is strictly decreasing, whereas *ψ*(*I*) is increasing since $\beta_{E}\left(\frac{w_{1}}{\alpha }I\right)$, *β*_*I*_(*I*), and $\beta_{V}\left(\frac{w_{1}\xi_{1}+\alpha \xi_{2}}{\sigma \alpha }I\right)$ are positive and decreasing functions of *I*. Additionally, one can easily verify that *ϕ*(0) = *S*_0_, *ϕ*(*I*_1_) = 0, where $I_{1}=\frac{\alpha \Lambda }{(\alpha +\mu )w_{1}}$, and

$$\psi (0)=(\alpha +\mu ){\left(\beta_{E}(0)+\frac{\alpha }{w_{1}}\beta_{I}(0)+\frac{w_{1}\xi_{1}+\alpha \xi_{2}}{\sigma w_{1}}\beta_{V}(0)\right)}^{-1}=\frac{S_{0}}{R_{0}}.$$

Thus, we conclude:

1. If $R_{0}>1$, these two curves have a unique intersection lying in the interior of ${\mathbb{R}}_{+}^{2}$, since *ψ*(0) < *ϕ*(0) and *ψ*(*I*_1_) ≥ *ψ*(0) > 0 = *ϕ*(*I*_1_). Furthermore, at this intersection point, Eq (2.6) yields a unique endemic equilibrium (EE)
  $$X_{*}=\left(S_{*},E_{*},I_{*},R_{*},V_{*}\right).$$
2. If $R_{0}\leq 1$, the two curves have no intersection in the interior of ${\mathbb{R}}_{+}^{2}$ as *ψ*(0) ≥ *ϕ*(0).

Therefore, by Eq (2.6), we find that the model (2.1) admits a unique equilibrium, the DFE *X*_0_, if $R_{0}\leq 1$; and it admits two equilibria, the DFE *X*_0_ and the EE X_*_, if $R_{0}>1$.

In what follows, we perform a study on the global stability of the DFE. By a simple comparison principle, we find that 0 ≤ *S* + *E* + *I* + *R* ≤ *S*_0_ and $0\leq V\leq \frac{\left(\xi_{1}+\xi_{2}\right)S_{0}}{\sigma }$. Thus, it leads to a biologically feasible domain

$$\Omega =\left\{(S,E,I,R,V)\in {\mathbb{R}}_{+}^{5}:S+E+I+R\leq S_{0},0\leq V\leq \frac{\left(\xi_{1}+\xi_{2}\right)S_{0}}{\sigma }\right\}.$$

Theorem 2.1. *The following statements hold for the model* (2.1).

1. *If* $R_{0}\leq 1$, *the DFE of system* (2.1) *is globally asymptotically stable in* Ω.
2. *If $R_{0}>1$, the DFE of system* (2.1) *is unstable and there exists a unique endemic equilibrium. Moreover, the disease is uniformly persistent in the interior of* Ω, *denoted by* $\overset{{}^{\circ}}{\Omega }$; namely, $\underset{t\to \infty }{\text{lim inf}}(E(t),I(t),V(t))>(\varepsilon ,\varepsilon ,\varepsilon )$ *for some ε* > 0.

*Proof.* Let **X** = (*E*, *I*, *V*)^*T*^. One can verify that

$$\frac{dX}{dt}\leq (F-V)X,$$

where the matrices *F* and *V* are given in Eq (2.3). By manipulating some algebraic computaion, we let **u** = (*β*_*E*_(0), *β*_*I*_(0), *β*_*V*_(0)). It then follows from the fact $R_{0}=\rho \left(FV^{-1}\right)=\rho \left(V^{-1}F\right)$ and direct calculation that **u** is a left eigenvector associated with the eigenvalue $R_{0}$ of the matrix *V*^−1^*F*; i.e., $uV^{-1}F=R_{0}u$. Consider a Lyapunov function

$$L_{0}=uV^{-1}X.$$

Differentiating L along the solutions of (2.1), we have

(2.9) $$\frac{dL_{0}}{dt}=uV^{-1}\frac{dX}{dt}\leq uV^{-1}(F-V)X=u\left(R_{0}-1\right)X.$$

If $R_{0}<1$, the equality $\frac{dL_{0}}{dt}=0$ implies that **uX** = 0. This leads to *E* = *I* = *V* = 0 by noting that all components of **u** are positive. Hence, when $R_{0}<1$, equations of (2.5) yield *S* = *S*_0_, and *E* = *I* = *R* = *V* = 0. Thus, the invariant set on which $\frac{dL_{0}}{dt}=0$ contains only the point *X*_0_.

If $R_{0}=1$, then the equality $\frac{dL_{0}}{dt}=0$ implies that

$$\left(\frac{\beta_{E}(E)S}{S_{0}}+\frac{\alpha \beta_{I}(I)}{w_{1}}+\frac{\left(w_{1}\xi_{1}+\alpha \xi_{2}\right)\beta_{V}(V)}{\sigma w_{1}}-\frac{\alpha +\mu }{S_{0}}\right)E+\left(\frac{S}{S_{0}}\beta_{I}(I)-\beta_{I}(0)\right)I+\left(\frac{S}{S_{0}}\beta_{V}(V)-\beta_{V}(0)\right)V=0.$$

It is easy to see that

$$\frac{S}{S_{0}}\beta_{I}(I)-\beta_{I}(0)\leq 0,\frac{S}{S_{0}}\beta_{V}(V)-\beta_{V}(0)\leq 0,$$

and

$$\frac{\beta_{E}(E)S}{S_{0}}+\frac{\alpha \beta_{I}(I)}{w_{1}}+\frac{\left(w_{1}\xi_{1}+\alpha \xi_{2}\right)\beta_{V}(V)}{\sigma w_{1}}-\frac{\alpha +\mu }{S_{0}}\leq \frac{\alpha +\mu }{S_{0}}\left(\frac{\beta_{E}(0)S_{0}}{\alpha +\mu }+\frac{\alpha \beta_{I}(0)S_{0}}{w_{1}(\alpha +\mu )}+\frac{\left(w_{1}\xi_{1}+\alpha \xi_{2}\right)\beta_{V}(0)S_{0}}{\sigma w_{1}(\alpha +\mu )}-1\right)=\frac{\alpha +\mu }{S_{0}}\left(R_{0}-1\right)=0.$$

Hence, we have either *E* = *I* = *V* = 0, or *β*_*E*_(*E*) = *β*_*E*_(0), *β*_*I*_(*I*) = *β*_*I*_(0), *β*_*V*_(*V*) = *β*_*V*_(0), and *S* = *S*_0_. As processed before, each of cases would indicate the DEF *X*_0_ is the only invariant set on $\{(S,E,I,R,V)\in \Omega :\frac{dL_{0}}{dt}=0\}$.

Therefore, when $R_{0}<1$ or $R_{0}=1$, the largest invariant set on which $\frac{dL_{0}}{dt}=0$ always consists of the singleton *X*_0_ = (*S*_0_, 0, 0, 0, 0). By LaSalle’s Invariant Principle [22], the DFE is globally asymptotically stable in Ω if $R_{0}\leq 1$.

In contrast, if $R_{0}>1$, then it follows from the continuity of the vector fields that $\frac{dL_{0}}{dt}>0$ in a neighborhood of the DFE in $\overset{{}^{\circ}}{\Omega }$. Thus the DFE is unstable by the Lyapunov stability theory. The last part of the theorem can be proved by the persistent theory [23] which is similar to the proof of Theorem 2.5 in Gao and Ruan [24]. □

In addition, we have conducted an analysis on the global asymptotic stability of the endemic equilibrium [25, 26], and the details are presented in the following theorem. Essentially, these stability results establish $R_{0}=1$ as a forward transcritical bifurcation point, or, a sharp threshold for disease dynamics, and indicate that reducing $R_{0}$ to values at or below unity will be sufficient to eradicate the disease. In other words, our model (2.1) exhibits *regular* threshold dynamics. In order to simplify our notations, we will adopt the abbreviations

$$\beta_{E}=\beta_{E}(E),\beta_{I}=\beta_{I}(I),\beta_{V}=\beta_{V}(V),\beta_{E}^{*}=\beta_{E}\left(E_{*}\right),\beta_{I}^{*}=\beta_{I}\left(I_{*}\right),\beta_{V}^{*}=\beta_{V}\left(V_{*}\right).$$

**Theorem 2.2**. *Assume that β*_*E*_(*E*)*E, β*_*I*_(*I*)*I and β*_*V*_(*V*)*V are non-decreasing functions of variables E, I and V, respectively. If* $R_{0}>1$, then the unique endemic equilibrium X_*_ *of system* (2.1) *is globally asymptotically stable in* $\overset{{}^{\circ}}{\Omega }$.

*Proof.* We let $L(y)=\int_{y_{*}}^{y}\frac{x-y_{*}}{x}dx$ for *y* > 0, where y_*_ > 0, and *y* can be replaced by *S*, *E*, *I*, or *V*. Clearly, *L*(*y*) ≥ 0 with the equality holding if and only if *y* = y_*_. Differentiating the four functions *L*(*S*), *L*(*E*), *L*(*I*), *L*(*V*) along the solution of system (2.1) and using the equilibrium equations yield

$$\frac{dL(S)}{dt}=\frac{S-S_{*}}{S}\frac{dS}{dt}\leq \frac{S-S_{*}}{S}\left(\beta_{E}^{*}S_{*}E_{*}-\beta_{E}SE+\beta_{I}^{*}S_{*}I_{*}-\beta_{I}SI+\beta_{V}^{*}S_{*}V_{*}-\beta_{V}SV\right)\;=\beta_{E}^{*}E_{*}S_{*}\left(1-\frac{S_{*}}{S}-\frac{\beta_{E}ES}{\beta_{E}^{*}E_{*}S_{*}}+\frac{\beta_{E}E}{\beta_{E}^{*}E_{*}}\right)+\beta_{I}^{*}I_{*}S_{*}\left(1-\frac{S_{*}}{S}-\frac{\beta_{I}IS}{\beta_{I}^{*}I_{*}S_{*}}+\frac{\beta_{I}I}{\beta_{I}^{*}I_{*}}\right)+\beta_{V}^{*}V_{*}S_{*}\left(1-\frac{S_{*}}{S}-\frac{\beta_{V}VS}{\beta_{V}^{*}V_{*}S_{*}}+\frac{\beta_{V}V}{\beta_{V}^{*}V_{*}}\right),$$

$$\frac{dL(E)}{dt}=\frac{E-E_{*}}{E}\frac{dE}{dt}=\frac{E-E_{*}}{E}\left(\beta_{E}SE+\beta_{I}SI+\beta_{V}SV-(\alpha +\mu )E\right)\;=\beta_{E}^{*}E_{*}S_{*}\left(\frac{\beta_{E}ES}{\beta_{E}^{*}E_{*}S_{*}}-\frac{E}{E_{*}}-\frac{\beta_{E}S}{\beta_{E}^{*}S_{*}}+1\right)+\beta_{I}^{*}I_{*}S_{*}\left(\frac{\beta_{I}IS}{\beta_{I}^{*}I_{*}S_{*}}-\frac{E}{E_{*}}-\frac{\beta_{I}ISE_{*}}{\beta_{I}^{*}I_{*}S_{*}E}+1\right)+\beta_{V}^{*}V_{*}S_{*}\left(\frac{\beta_{V}VS}{\beta_{V}^{*}V_{*}S_{*}}-\frac{E}{E_{*}}-\frac{\beta_{V}VSE_{*}}{\beta_{V}^{*}V_{*}S_{*}E}+1\right).$$

Hence,

$$\frac{dL(S)}{dt}+\frac{dL(E)}{dt}\leq \beta_{E}^{*}E_{*}S_{*}\left(2-\frac{S_{*}}{S}-\frac{E}{E_{*}}+\frac{\beta_{E}E}{\beta_{E}^{*}E_{*}}-\frac{\beta_{E}S}{\beta_{E}^{*}S_{*}}\right)\;+\beta_{I}^{*}I_{*}S_{*}\left(2-\frac{S_{*}}{S}-\frac{E}{E_{*}}+\frac{\beta_{I}I}{\beta_{I}^{*}I_{*}}-\frac{\beta_{I}ISE_{*}}{\beta_{I}^{*}I_{*}S_{*}E}\right)\;+\beta_{V}^{*}V_{*}S_{*}\left(2-\frac{S_{*}}{S}-\frac{E}{E_{*}}+\frac{\beta_{V}V}{\beta_{V}^{*}V_{*}}-\frac{\beta_{V}VSE_{*}}{\beta_{V}^{*}V_{*}S_{*}E}\right)\;\leq \beta_{E}^{*}E_{*}S_{*}\left(\frac{\beta_{E}E}{\beta_{E}^{*}E_{*}}-1\right)\left(1-\frac{\beta_{E}^{*}}{\beta_{E}}\right)\;+\beta_{I}^{*}I_{*}S_{*}\left\{\left(\frac{\beta_{I}I}{\beta_{I}^{*}I_{*}}-1\right)\left(1-\frac{\beta_{I}^{*}}{\beta_{I}}\right)+\frac{I}{I_{*}}-\frac{E}{E_{*}}-\text{ln}\frac{I}{I_{*}}+\text{ln}\frac{E}{E_{*}}\right\}\;+\beta_{V}^{*}V_{*}S_{*}\left\{\left(\frac{\beta_{V}V}{\beta_{V}^{*}V_{*}}-1\right)\left(1-\frac{\beta_{V}^{*}}{\beta_{V}}\right)+\frac{V}{V_{*}}-\frac{E}{E_{*}}-\text{ln}\frac{V}{V_{*}}+\text{ln}\frac{E}{E_{*}}\right\}\;\leq \beta_{I}^{*}I_{*}S_{*}\left(\frac{I}{I_{*}}-\frac{E}{E_{*}}-\text{ln}\frac{I}{I_{*}}+\text{ln}\frac{E}{E_{*}}\right)+\beta_{V}^{*}V_{*}S_{*}\left(\frac{V}{V_{*}}-\frac{E}{E_{*}}-\text{ln}\frac{V}{V_{*}}+\text{ln}\frac{E}{E_{*}}\right).$$

The last inequality follows from the assumptions that *β*_*P*_(*P*) and *β*_*P*_(*P*)*P*, where *P* can represent *E*, *I*, or *V*, are non-increasing and non-decreasing functions of *P*, respectively. This implies

$$1-\frac{\beta_{p}^{*}}{\beta_{P}}\leq 0\Leftrightarrow P^{*}\leq P\Leftrightarrow \frac{\beta_{P}P}{\beta_{p}^{*}P_{*}}-1\geq 0.$$

Similarly, one can verify that

$$\frac{dL(I)}{dt}=\alpha E_{*}\left(\frac{E}{E_{*}}-\frac{I}{I_{*}}-\frac{I_{*}E}{IE_{*}}+1\right)\leq \alpha E_{*}\left(\frac{E}{E_{*}}-\frac{I}{I_{*}}+\text{ln}\frac{I}{I_{*}}-\text{ln}\frac{E}{E_{*}}\right),$$

$$\frac{dL(V)}{dt}=\xi_{1}E_{*}\left(\frac{E}{E_{*}}-\frac{V}{V_{*}}-\frac{V_{*}E}{VE_{*}}+1\right)+\xi_{2}I_{*}\left(\frac{I}{I_{*}}-\frac{V}{V_{*}}-\frac{V_{*}I}{VI_{*}}+1\right)\leq \xi_{1}E_{*}\left(\frac{E}{E_{*}}-\frac{V}{V_{*}}+\text{ln}\frac{V}{V_{*}}-\text{ln}\frac{E}{E_{*}}\right)+\xi_{2}I_{*}\left(\frac{I}{I_{*}}-\frac{V}{V_{*}}+\text{ln}\frac{V}{V_{*}}-\text{ln}\frac{I}{I_{*}}\right).$$

Let $c_{1}=\frac{\beta_{I}^{*}I_{*}S_{*}}{\alpha E_{*}}+\frac{\xi_{2}\beta_{V}^{*}V_{*}S_{*}}{\left(w_{1}\xi_{1}+\alpha \xi_{2}\right)E_{*}}$ and $c_{2}=\frac{w_{1}\beta_{V}^{*}V_{*}S_{*}}{\left(w_{1}\xi_{1}+\alpha \xi_{2}\right)E_{*}}$. Then, we claim that

$$L_{1}=L(S)+L(E)+c_{1}L(I)+c_{2}L(V)$$

is a Lyapunov function for system (2.1). Clearly, $L_{1}\geq 0$ and

$$\frac{dL}{dt}\leq \left(\beta_{I}^{*}I_{*}S_{*}+\beta_{V}^{*}V_{*}S_{*}-c_{1}\alpha E_{*}-c_{2}\xi_{1}E_{*}\right)\left(\text{ln}\frac{E}{E_{*}}-\frac{E}{E_{*}}\right)+\left(\beta_{I}^{*}I_{*}S_{*}-c_{1}\alpha E_{*}+c_{2}\xi_{2}I_{*}\right)\left(\frac{I}{I_{*}}-\text{ln}\frac{I}{I_{*}}\right)+\left(\beta_{V}^{*}V_{*}S_{*}-c_{2}\left(\xi_{1}E_{*}+\xi_{2}I_{*}\right)\right)\left(\frac{V}{V_{*}}-\text{ln}\frac{V}{V_{*}}\right)\text{=0}$$

with the equality holding if and only if (*S*, *E*, *I*, *V*) = (S_*_, E_*_, I_*_, V_*_). Thus, one can easily see that the largest invariant set where $\frac{dL}{dt}=0$ is the singleton {X_*_ = (S_*_, E_*_, I_*_, R_*_, V_*_)}. Therefore, X_*_ is globally asymptotically stable in $\overset{{}^{\circ}}{\Omega }$. □

## 3. Numerical results

We now apply our model to study the COVID-19 epidemic in the city of Wuhan. We use the outbreak data published daily by WHO and other sources [7, 27–30]. These data sets contain the daily reported new cases, cumulative cases, and disease-caused deaths for the city of Wuhan, as well as each province in China and all other countries that have reported COVID-19 infection.

To conduct the numerical simulation, we consider the following functions for the three transmission rates in our model:

(3.1) $$\beta_{E}(E)=\frac{\beta_{E0}}{1+cE},\;\beta_{I}(I)=\frac{\beta_{I0}}{1+cI},\;\beta_{V}(V)=\frac{\beta_{V0}}{1+cV},$$

where *β*_*E*0_, *β*_*I*0_ and *β*_*V*0_ (all positive constants) denote the maximum values of these transmission rates, and *c* is a positive coefficient providing adjustment to the (otherwise constant) transmission rates. For example, *β*_*I*_(*I*) achieves its maximum when *I* = 0, with *β*_*I*_(0) = *β*_*I*0_, and *β*_*I*_(*I*) decreases when *I* is increasing. Note also that *β*_*E*_(*E*)*E*, *β*_*I*_(*I*)*I* and *β*_*V*_(*V*)*V* are non-decreasing functions which satisfy the assumption of Theorem 2.2.

We implement our model and conduct numerical simulation for an epidemic period starting from January 23, 2020, when the city of Wuhan was placed in quarantine, to February 10, 2020. According to the estimate made by the Chinese government, about 9 million people remain in Wuhan after January 23 and they are not allowed to move out of the city. Meanwhile, only a relatively small number of people (mainly public health professionals) travel into the city since its lockdown. Thus, the influx rate Λ in our model is only based on newborns in Wuhan. The values of the transmission constants *β*_*E*0_ and *β*_*I*0_ can be found in a recent study [13]. The incubation period of the infection ranges between 2–14 days, with a mean of 5–7 days [31], and we take the value of 7 days in our model. The average recovery period is about 15 days [31], and so we set the disease recovery rate as *γ* = 1/15 per day. Members of the coronavirus family can survive in the environment from a few hours to several days [19], and we take the value of 1 day which results in a virus removal rate *σ* = 1 per day. Additionally, since the Chinese government has been implementing a very strict isolation policy and intense medical care for all the confirmed cases, represented by *I* in our model, the chance of those infected individuals spreading the coronavirus to the environment connected with the general public is very low, and so we assume the virus shedding rate from the infected individuals is zero; i.e., *ξ*_2_ = 0. Note that our results in Theorem 2.1 and Theorem 2.2 still hold in this case since the contribution of the coronavirus to the environmental reservoir remains a positive number *w*_1_*ξ*_1_. These and other parameters, their values and sources are provided in Table 1. There are three parameters, however, that remain to be determined: the environment-to-human transmission constant *β*_*V*0_, the transmission adjustment coefficient *c*, and the virus shedding rate *ξ*_1_ by the exposed individuals. The values of these parameters are not available in the literature because the models published thus far have not considered the environmental component for the COVID-19 infection, and they have generally applied constant transmission rates which remain fixed in time.

**Figure 1.**
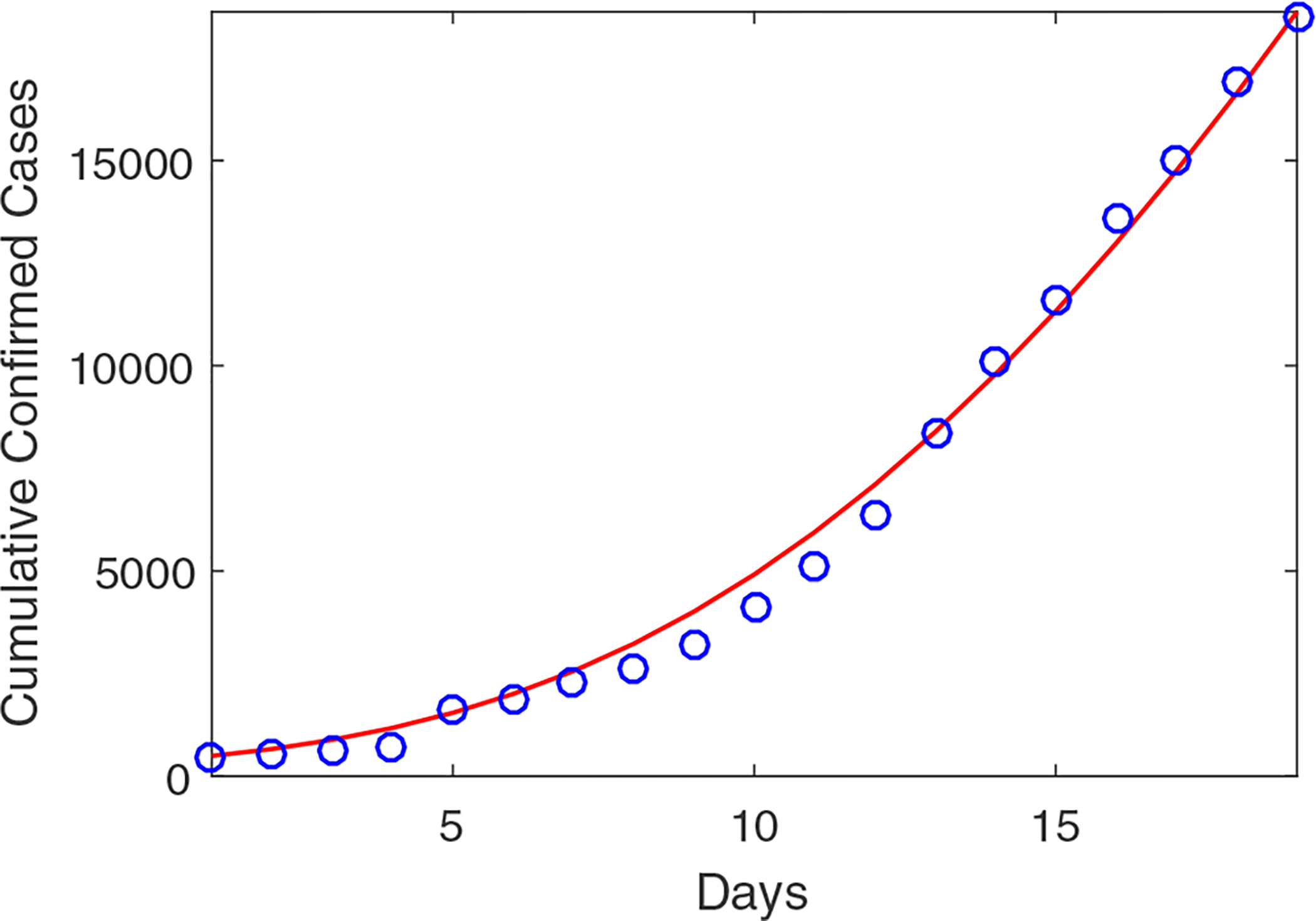
Cumulative confirmed cases for the city of Wuhan from January 23, 2020 to February 10, 2020. Circles (in blue) denote the reported cases and solid line (in red) denotes the simulation result. The basic reproduction number is $R_{0}=4.25$ based on the parameters from Table 1 and the result of data fitting.

To estimate the values for these three parameters, similar to [32], we fit our model to the daily reported infection data for Wuhan from January 23 to February 10 by using the standard least squares method. Based on reported data, the initial condition is set as (*S*(0), *E*(0), *I*(0), *R*(0), *V*(0)) = (89985051000, 475, 10, 10000) [33]. Figure 1 shows the numbers of cumulative confirmed cases in Wuhan during this period versus our fitting curve. The parameter values and their 95% confidence intervals are presented in Table 2. The normalized mean square error (NMSE) for the data fitting is found as 0.0058.

Based on the parameter values from data fitting, we are able to evaluate the basic reproduction number $R_{0}=4.25$. Specifically, we find that

$$R_{1}=1.959,\;R_{2}=0.789,\;R_{3}=1.497,$$

which quantify the infection risk from each of the three transmission routes. Among these three components, the largest one $\left(R_{1}\right)$ comes from the exposed-to-susceptible transmission, since exposed individuals show no symptoms and can easily spread the infection to other people with close contact, often in an unconscious manner. Meanwhile, the smallest component $\left(R_{2}\right)$ comes from the infected-to-susceptible transmission, possibly due to the strict isolation policy on the symptomatic infected individuals. In addition, we observe that $R_{3}=1.497$, showing a significant contribution from the environmental reservoir toward the overall infection risk.

**Figure 2.**
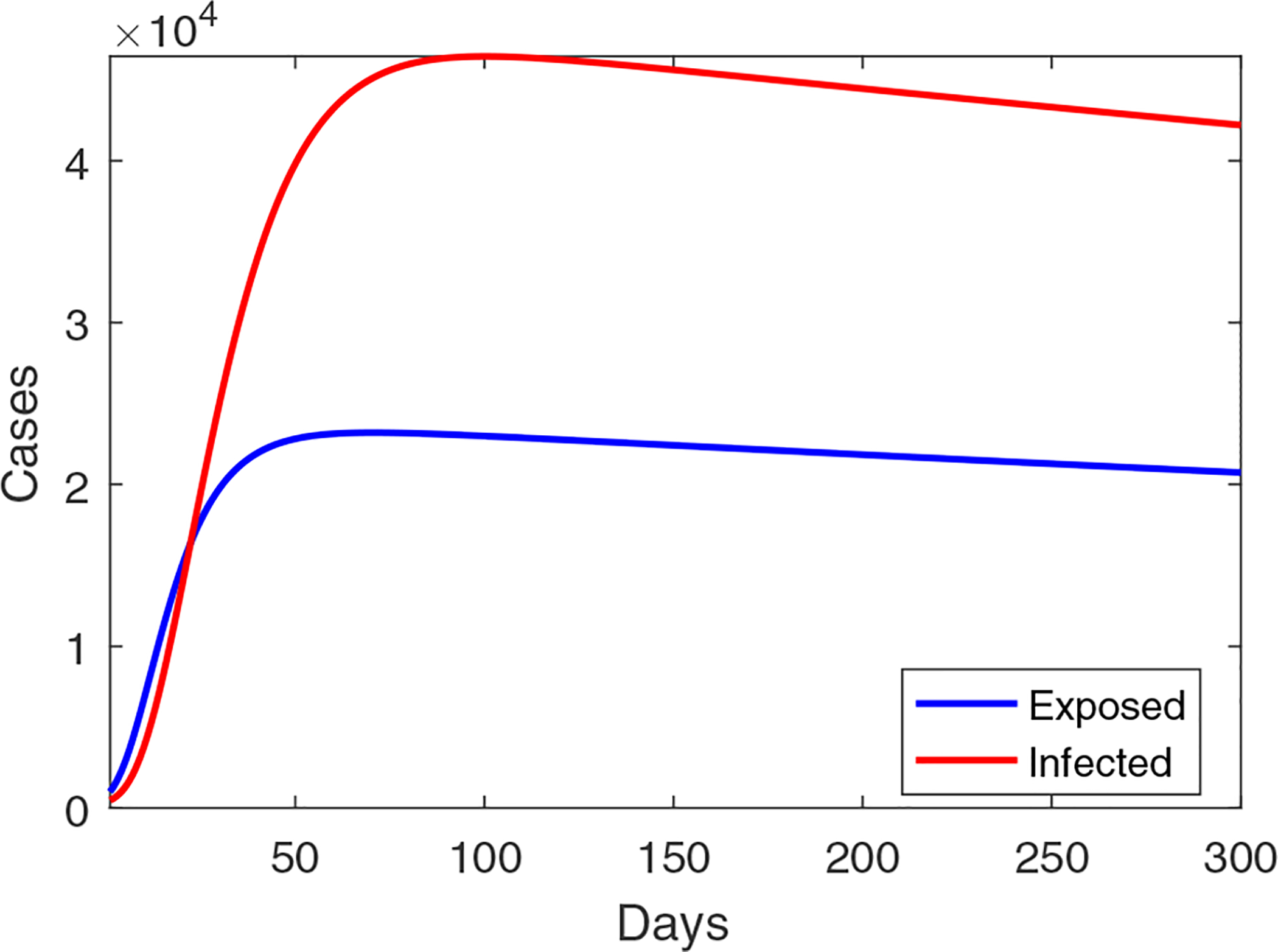
A simulation result for the outbreak size in Wuhan using the transmission rates formulated in Eq (3.1), the parameters from Table 1, and the result of data fitting.

**Figure 3.**
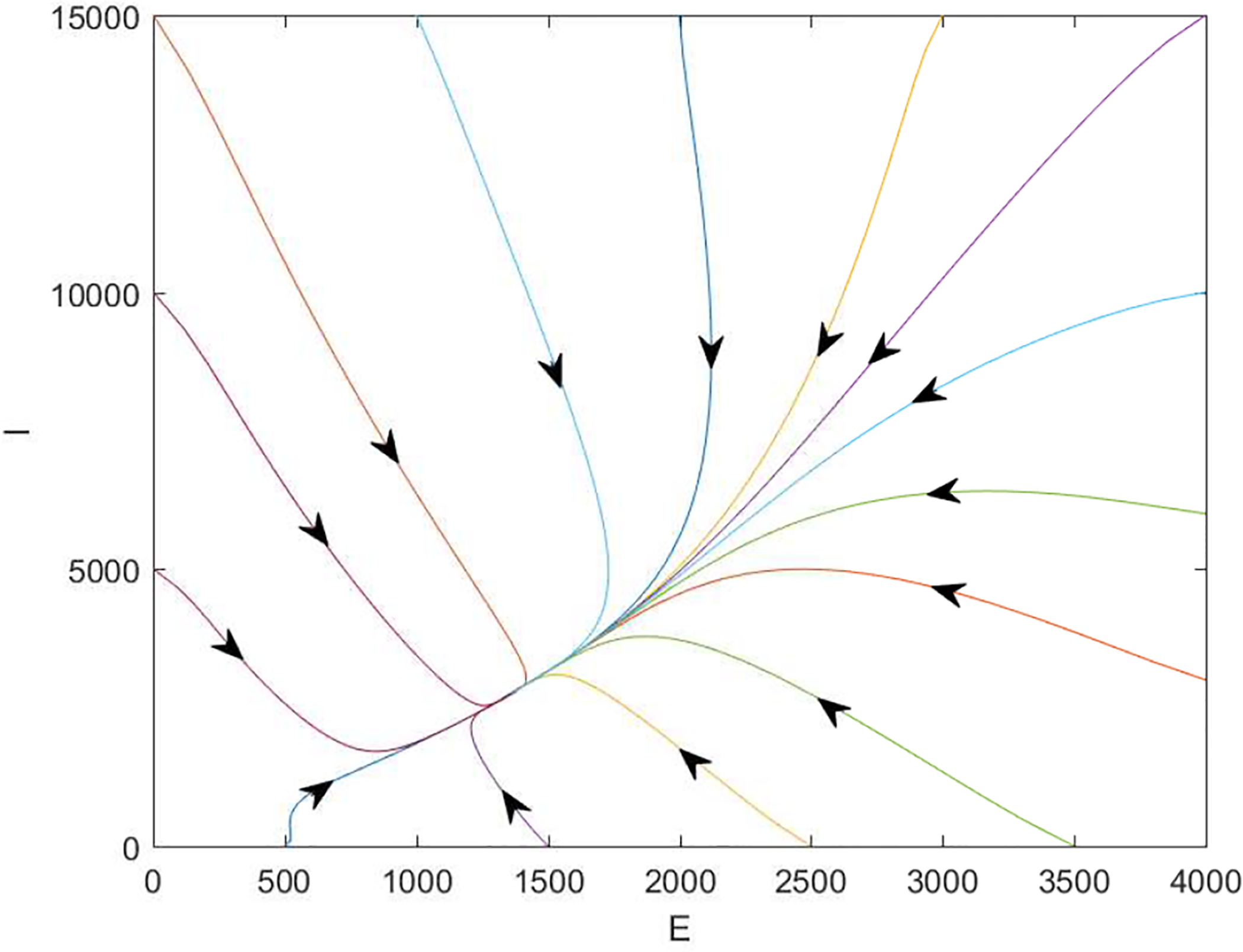
A typical phase portrait for *I* vs. *E* with $R_{0}=4.25$. Every orbit starts with a different initial point and converges to the endemic equilibrium where (E_*_, I_*_) = (1353, 2735).

Figure 2 displays a short-term prediction for *I* (the infected individuals) and *E* (the exposed individuals) in Wuhan using our model. It shows that the infection level, starting from January 23 (marked as day 0 in our simulation), would continue increasing for about 80 days, reach a peak value around 45,000 infections, and then gradually go down afterwards. Meanwhile, the long-term behavior of the epidemic would be determined by the property of the endemic equlibrium of the system, which is found as X_*_ = (2583683, 1353, 2735, 6528015, 3111). A phase portrait of *I* vs. *E* is provided in Figure 3, where all the solution orbits converge to the endemic equlibrium, illustrating its global asymptotical stability that is stated in Theorem 2.2.

**Figure 4.**
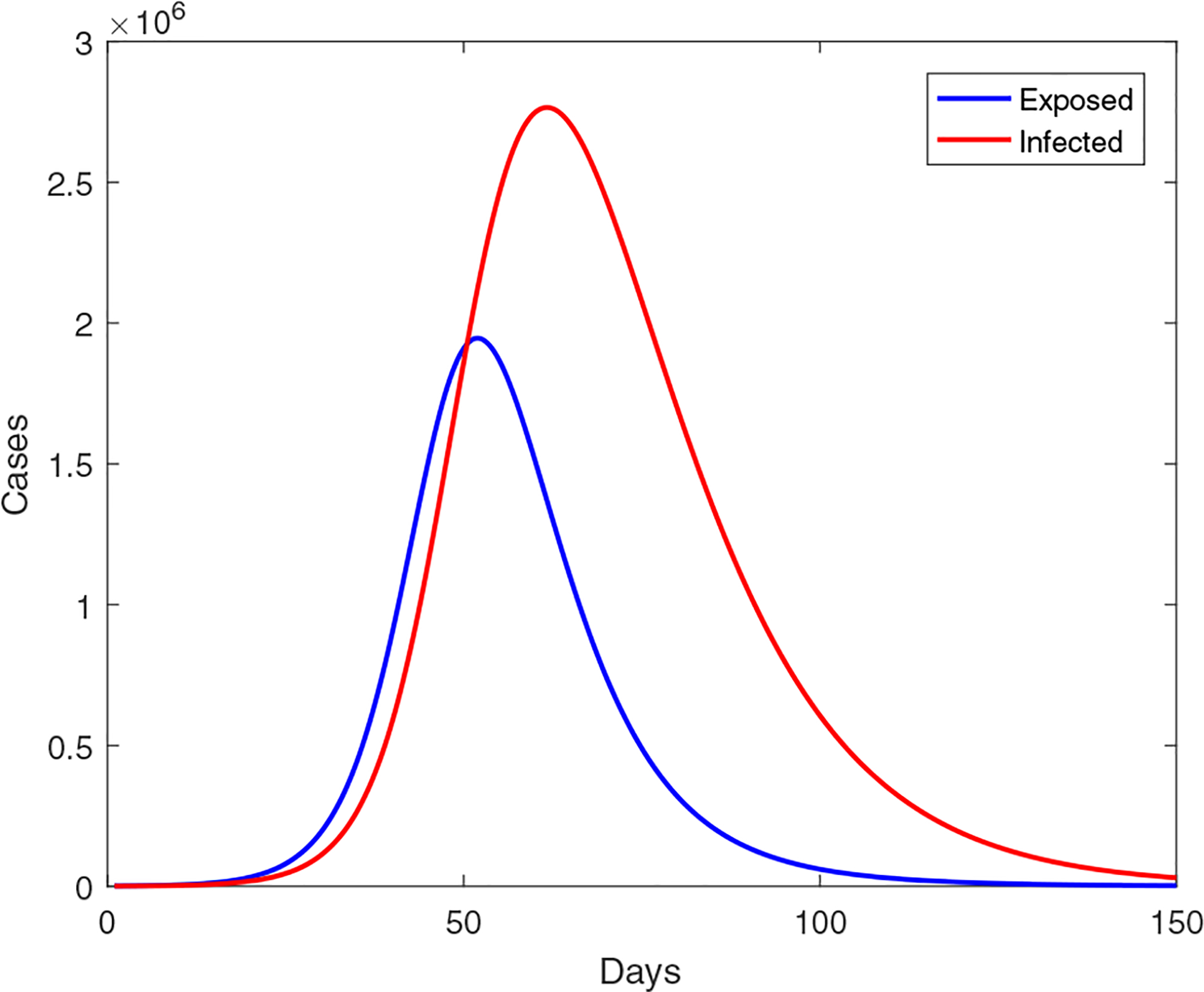
A simulation result for the outbreak size in Wuhan using the constant transmission rates given in Eq (3.2), the parameters from Table 1, and the result of data fitting.

In addition, we have performed a numerical test using simple, constant transmission rates in our model:

(3.2) $$\beta_{E}(E)=\beta_{E0},\;\beta_{I}(I)=\beta_{I0},\;\beta_{V}(V)=\beta_{V0},$$

equivalent to setting *c* = 0 in Eq (3.1). This leaves two parameters, *β*_*V*0_ and *ξ*_1_, to be estimated by data fitting. Using the same set of data, we find that *ξ*_1_ ≈ 4.28 with the 95% confidence interval (0, 15.611), and *β*_*V*0_ ≈ 4.91 × 10^−10^ with the 95% confidence interval (4.218 × 10^−10^, 5.603 × 10^−10^). The normalized mean square error (NMSE) for the data fitting is 0.0266, larger than that in the previous scenario, 0.0058. Meanwhile, Figure 4 shows a prediction of the Wuhan coronavirus outbreak size in this setting. Compared to Figure 2, We now clearly observe a significantly higher level of infection; particularly, the peak value appears at 2.8 × 10^6^, which is extremely large and clearly unrealistic. The result demonstrates that using fixed transmission rates, which do not take into account the strong disease control measures currently on-going in Wuhan, may overestimate the epidemic severity and generate misguided information.

## 4. Discussion

We have proposed a mathematical model to investigate the on-going novel coronavirus epidemic in Wuhan, China. There are two unique features in our model: (1) the incorporation of an environmental reservoir into the disease transmission dynamics, and (2) the use of non-constant transmission rates which change with the epidemiological status and environmental conditions and which reflect the impact of the disease control measures implemented in Wuhan. We have conducted a detailed analysis of this model, and applied it to study the Wuhan epidemic using publicly reported data.

The basic reproduction number $R_{0}$ of this model consists of three parts, representing the three different transmission routes; i.e., from the exposed individuals, the infected individuals, and the environmental reservoir, to the susceptible individuals. These three transmission modes collectively shape the overall disease risk of this epidemic, suggesting that intervention strategies should target all these three transmission routes. Our equilibrium analysis of this model shows that the disease dynamics exhibit a regular threshold at $R_{0}=1$. We have established the global asymptotic stability of the disease-free equilibrium when $R_{0}<1$, and the global asymptotic stability of the endemic equilibrium when $R_{0}>1$.

Our numerical simulation results demonstrate the application of our model to the COVID-19 outbreak in Wuhan. Our model can fit the reported data well. Through data fitting, we obtain an estimate of basic reproduction number, $R_{0}=4.25$. In particular, we find that the contribution of the environmental reservoir (measured by $R_{3}$) is significant in shaping the overall disease risk. Our model predicts the appearance of an epidemic peak, after which the infection level would decrease and approach an endemic state in the long run. We also find that if we use constant transmission rates instead, the model would predict a much higher and unrealistic epidemic peak. This is caused by the fixed transmission rates that do not reflect the impact of on-going disease control measures. It is an indication that using epidemiologically and environmentally dependent transmission rates can potentially generate more practical simulation results.

At present, many aspects regarding the pathology, ecology and epidemiology of the novel coronavirus remain unknown, which adds challenges to the mathematical modeling. Particularly, in our current model, we have employed a bilinear incidence rate based on the law of mass action to represent the environment-to-human transmission route [34]. Practically, though, a saturation based incidence rate might better characterize the environmental pathogen, and we hope to investigate it in our future modeling efforts. Meanwhile, the transmission rates *β*_*E*_ and *β*_*V*_ in our model depend on *E* and *V*, respectively, while in reality the exposed population *E* and the environmental pathogen concentration *V* may be unknown. To better quantify these transmission rates, we could instead assume that they are functions of *I*; i.e., *β*_*E*_(*I*) and *β*_*V*_(*I*), since the infected population *I* can be easily calculated from the reported data. Nevertheless, it is reasonable to assume that *E* and *V* are positively correlated to *I*, and so the qualitative properties of these transmission functions would remain the same under both formulations.

Given the current development of COVID-19, it is widely speculated that this disease would persist in the human world and become endemic. Our mathematical analysis and numerical simulation results support this speculation. The findings in this study imply that we should be prepared to fight the coronavirus infection for a much longer term than that of the current epidemic wave, in order to reduce the endemic burden and potentially eradicate the disease eventually. Among other intervention strategies, new vaccines for the novel coronavirus, which are currently in research and development, could play an important role in achieving that goal.

We emphasize that our data fitting is based on the reported confirmed cases in Wuhan from January 23 to February 10 in 2020. These confirmed cases were determined by the method of nucleic acid testing kits. On February 12, 2020, the national health commission in China started including cases confirmed by another method; i.e, clinical diagnosis, which refers to using CT imaging scans to diagnose patients. This change of criteria led to a surge of confirmed cases on February 12 (with an increase of about 14,000 new cases for Wuhan in a single day), and our current study does not take into account this factor. In this regard, our prediction of the epidemic duration and size should be interpreted as applicable only to the confirmed cases based on the previous, more strict, testing method. The issues regarding the accuracy, reliability and standard of reported data are complex and are beyond the scope of this work, which is more oriented on the mathematical modeling side. We plan to address the new development of the outbreak data in another piece of work in the near future. We also plan to expand our modeling efforts to the province and country levels beyond the epicenter, the city of Wuhan, and study the spread of the novel coronavirus in larger spatial scales.

## Figures and Tables

**Table 1.** Definitions and values of model parameters.

Parameter	Definition	Estimated mean value	Source
Λ	Influx rate	271.23 per day	[30]
*β*_*E*0_	Transmission constant between *S* and *E*	3.11 × 10^−8^/person/day	[13]
*β*_*I*0_	Transmission constant between *S* and *I*	0.62 × 10^−8^/person/day	[13]
*β*_*V*0_	Transmission constant between *S* and *V*	fitting by data	-
*c*	Transmission adjustment coefficient	fitting by data	-
*μ*	Natural death rate	3.01 × 10^−5^ per day	[30]
1/*α*	Incubation period	7 days	[31]
*w*	Disease-induced death rate	0.01 per day	[30]
*γ*	Recovery rate	1/15 per day	[31]
*σ*	Removal rate of virus	1 per day	[19]
*ξ*_1_	Virus shedding rate by exposed people	fitting by data	-
*ξ*_2_	Virus shedding rate by infected people	0 per person per day per ml	-

**Table 2.** Parameter estimates from data fitting.

Parameter	Fitting value	95% Confidence Interval
*ξ*_1_	2.30	(0, 23.114)
*β*_*V*0_	1.03 × 10^−8^	(0, 5.074 × 10^−8^)
*C*	1.01 × 10^−4^	(0.585 × 10^−4^, 1.426 × 10^−4^)
